# Outcomes of axitinib versus sunitinib as first‐line therapy to patients with metastatic renal cell carcinoma in the immune‐oncology era

**DOI:** 10.1002/cam4.4130

**Published:** 2021-07-27

**Authors:** Kazuyuki Numakura, Yumin Muto, Sei Naito, Shingo Hatakeyama, Renpei Kato, Tomoyuki Koguchi, Takahiro Kojima, Yoshihide Kawasaki, Syuya Kandori, Sadafumi Kawamura, Yoichi Arai, Akihiro Ito, Hiroyuki Nishiyama, Yoshiyuki Kojima, Wataru Obara, Chikara Ohyama, Norihiko Tsuchiya, Tomonori Habuchi

**Affiliations:** ^1^ Department of Urology Akita University Graduate School of Medicine Akita Japan; ^2^ Department of Urology Yamagata University Faculty of Medicine Yamagata Japan; ^3^ Department of Urology Hirosaki University Graduate School of Medicine Hirosaki Japan; ^4^ Department of Urology Iwate Medical University Morioka Japan; ^5^ Department of Urology Fukushima Medical University Fukushima Japan; ^6^ Department of Urology and Andrology Tsukuba University Graduate School of Comprehensive Human Sciences Tsukuba Japan; ^7^ Department of Urology Tohoku University Graduate School of Medicine Tohoku Japan; ^8^ Department of Urology Miyagi Cancer Center Natori Japan

**Keywords:** axitinib, metastatic renal cell carcinoma, nivolumab

## Abstract

Although combination immune checkpoint inhibitor (immuno‐oncology [IO]) therapy is the first‐line treatment for metastatic renal cell carcinoma (mRCC), it mostly causes resistance and tumor regrowth. Therefore, an optimal second‐line therapy is necessary. Such therapy typically comprises vascular endothelial growth factor receptor‐tyrosine kinase inhibitors (VEGFR‐TKIs). This study was aimed at comparing the efficacy of two TKIs—axitinib and sunitinib—in mRCC patients. From January 2008 to October 2018, we registered 703 mRCC patients from 8 Japanese institutes. Of these, 408 patients received axitinib or sunitinib as the first‐line treatment. Thereafter, efficacy and survival rate were compared between the axitinib and sunitinib groups. To reduce the effects of selection bias and potential confounders, propensity score matching analysis was performed. Axitinib and sunitinib were administered in 274 and 134 patients, respectively. More than 25% of the patients received nivolumab sequence therapy. To calculate the propensity scores for each patient, we performed multivariate logistic regression analysis. The objective response rate, progression‐free survival (PFS), cause‐specific survival, and overall survival (OS) were significantly better in the axitinib group than in the sunitinib group. Furthermore, the OS was better in the nivolumab‐treated patients in the axitinib group. Axitinib showed higher efficacy and afforded greater survival benefits than did sunitinib when administered as first‐line therapy in mRCC patients. Thus, from among VEGFR‐TKIs, axitinib might be a possible option for application in the middle of IO drug‐based treatment sequences.

## Graphical abstract

Compared to sunitinib, axitinib showed higher efficacy and afforded greater survival benefits as first‐line therapy in patients with metastatic renal cell carcinoma. Axitinib might be better than sunitinib for application in the middle of IO drug‐based treatment sequences.

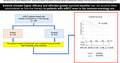

**Abbreviations**
CI: confidence interval
CR: complete response
CSS: cancer‐specific survival
ECOG: Eastern Cooperative Oncology Group
HR: hazard ratio
IMDC: International Metastatic Renal Cell Carcinoma Database Consortium
IO: immuno‐oncology
mRCC: metastatic renal cell carcinoma
OR: odds ratio
OS: overall survival
PD: progressive disease
PFS: progression‐free survival
PS: performance status
RCC: renal cell carcinoma
VEGFR‐TKI: vascular endothelial growth factor receptor‐tyrosine kinase inhibitors

## 1 INTRODUCTION

At the time of the first diagnosis, 20%–30% of all patients with renal cell carcinoma (RCC) already have systemic disease.^1^ In the last decade, vascular endothelial growth factor receptor‐tyrosine kinase inhibitors (VEGFR‐TKIs) have become the standard of care for metastatic RCC (mRCC).^2^, ^3^ Furthermore, immuno‐oncology (IO) agents, which block immune checkpoints and restore tumor‐specific T‐cell‐mediated immune responses, have changed the treatment paradigm for mRCC. IO combination therapies as first‐line therapy have shown promising early results for mRCC.^4^, ^5^, ^6^ However, there is a risk of primary refractory status and subsequent resistance and regrowth after IO combination therapies in many patients.^7^, ^8^ Moreover, IO combination therapy has not shown a clear advantage over VEGFR‐TKI therapy in patients with mRCC that have favorable International Metastatic Renal Cell Carcinoma Database Consortium (IMDC) risk scores.^4^, ^5^, ^6^ Hence, an optimal selection of first‐, second‐, or later‐line therapies is under debate. In this regard, VEGFR‐TKIs are thought to be the mainstay treatments, while IO drugs remain the standard treatment.^9^ This study was aimed at comparing the clinical efficacy of axitinib and sunitinib used in Japanese patients with mRCC for elucidating an optimal VEGFR‐TKI in the IO era.

## 2 MATERIALS AND METHODS

From January 2008 to October 2018, 703 patients with mRCC from 8 Japanese institutions (Michinoku RCC) were retrospectively included in this study. Of these patients, 408 were treated with axitinib or sunitinib as the first‐line treatment (Figure S1). Clinical efficacy and survival rate were comparatively evaluated between the axitinib and sunitinib groups. To reduce the effects of selection biases and potential confounders in this observational study, propensity score matching analysis and Cox hazard regression model were applied.

### 2.1 Eligibility criteria

Patients with histologically proven mRCC regardless of Eastern Cooperative Oncology Group (ECOG) performance status (PS) were included in this study. This study was approved by all eight institutional review boards. All procedures were performed according to the tenets of the 1964 Declaration of Helsinki. Informed consent was received for all the participating patients.

### 2.2 Objective

The primary objective was to compare patients’ survival rates, including progression‐free survival (PFS), cancer‐specific survival (CSS), and OS rates between mRCC patients treated with axitinib and sunitinib as first‐line therapy. CSS and OS were also compared between the groups who treated by nivolumab sequentially.

### 2.3 Treatment and follow‐up examinations

The following determinations were made before starting treatment and repeated during therapy based on the attending physician's decision: complete medical history, physical examination, ECOG PS, blood cell counts with differential and platelet counts, biochemical profile (including electrolytes, renal and hepatic function, coagulation, pancreatic amylase, and lipase), urinalyses, and chest radiography. Some potential prognostic markers (c‐reactive protein, neutrophil–lymphocyte ratio, and alkaline phosphatase) were also measured.^10^, ^11^, ^12^ Tumor response was evaluated using the Response Evaluation Criteria in Solid Tumors version 1.1.

### 2.4 Statistical analysis

Progression‐free survival was defined as the time between the initiation of VEGFR‐TKI treatment and disease progression or death as confirmed using radiological images or based on obvious clinical manifestations of progressive disease. CSS was defined as the time between the initiation of VEGFR‐TKI treatment and death due to cancer. OS was defined as the time between the initiation of VEGFR‐TKI treatment and death. The database record was closed upon patient death or the final follow‐up. Data are expressed as the median and range, and differences with a *p* value <0.05 were considered to be statistically significant. The chi‐square test was used to examine differences and calculate the odds ratio (OR) for categorical data. PFS and OS were stratified using the Kaplan–Meier method. The Cox proportional hazard regression model was used for the analysis of hazard ratio (HR) and 95% confidence interval (CI). Data were analyzed using SPSS version 26.0 statistical software (SPSS Japan Inc.). To reduce the effects of selection biases and potential confounders, we performed a propensity score matching analysis. Propensity scores were calculated for each patient using multivariate logistic regression analysis with the following covariates: sex, age, histology, prior nephrectomy, nivolumab as sequential therapy, clinical stage at the first diagnosis, and the IMDC score.

## 3 RESULTS

### 3.1 Patient characteristics

This study included 703 patients who were diagnosed with advanced RCC and treated with systemic therapies at 8 institutes between January 2008 and August 2018 (Michinoku RCC database). Of these patients, 408 patients who received axitinib or sunitinib as first‐line therapy were analyzed. The median patient age was 66 (range: 24–89) years. The median duration of VEGFR‐TKI therapy was 20 (range: 1–144) months. All patients were Japanese, and the cohort included 303 (74.3%) men and 105 (25.7%) women. As first‐line systemic therapy, 134 and 274 patients received axitinib and sunitinib, respectively. The characteristics of the two groups were comparable (Table 1). Axitinib was administered to patients who were elderly, less receiving a nephrectomy, and had a higher IMDC score compared to the patients to whom sunitinib was administered. In this cohort, 108 patients (26.5%) were administered nivolumab sequentially (the detail of these characters is shown in Table S1). No significant difference was found between the groups regarding factors including body mass index (BMI), histology, tumor grade, clinical stage, number of metastatic sites, sequential nivolumab therapy, level of c‐reactive protein, and neutrophil–lymphocyte ratio.

**TABLE 1:** Patients characteristics

	All patients (*N* = 408)	Axitinib (*N* = 134)	Sunitinib (*N* = 274)	*p*
Age				
Median year (range)	66 (24–89)	69 (24–89)	65 (18–85)	<0.001
BMI				
Median kg/m^2^ (range)	22.4 (14.4–46.6)	22.2 (15.4–36.6)	22.5 (14.4–46.6)	0.634
Sex, *n* (%)				
Male: Female	303 (74): 105 (26)	94 (70): 40 (30)	209 (76): 65 (24)	0.187
Nephrectomy, *n* (%)				
Yes: No	274 (67): 134 (33)	80 (60): 54 (40)	194 (71): 80 (29)	0.033
Histology, *n* (%)				
Clear cell	332 (81)	111 (83)	221 (81)	0.880
With spindle compornent	61 (15)	21 (16)	40 (15)	
Papillary	15 (4)	5 (4)	10 (4)	
Others	31 (8)	13 (10)	18 (7)	
Unknown	30 (7)	5 (4)	25 (9)	
Grade, *n* (%)				
1	7 (2)	2 (1)	5 (2)	0.834
2	112 (27)	42 (31)	70 (26)	
3	166 (41)	65 (49)	101 (37)	
Unknown	123 (30)	25 (61)	98 (36)	
Clinical stage, *n* (%)				
1	42 (10)	15 (11)	27 (10)	0.961
2	30 (7)	11 (8)	19 (7)	
3	67 (16)	23 (17)	44 (16)	
4	259 (63)	85 (63)	174 (64)	
Unknown	10 (2)	0 (0)	10 (4)	
IMDC risk classification, *n* (%)				
Favorable	31 (8)	8 (6)	23 (8)	0.012
Intermediate	181 (44)	52 (39)	129 (47)	
Poor	132 (32)	58 (43)	74 (27)	
Unclassified	64 (16)	16 (12)	48 (18)	
Metastatic site, *n* (%)				
1	173 (42)	63 (47)	110 (40)	0.157
2	130 (32)	36 (27)	94 (34)	
3≤	94 (23)	33 (25)	61 (22)	
Unknown	11 (3)	9 (7)	2 (1)	
Nivolumab was used in seaquential therapy, *n* (%)				
Yes: No	108 (26): 300 (74)	33 (25): 101 (75)	75 (27): 199 (73)	0.633
CRP				
Median (range)	0.7 (0–25.5)	0.9 (0–24.8)	0.7 (0–25.5)	0.402
NLR				
3.6≤	135 (33)	48 (36)	87 (32)	0.565
ALP				
Higher than institutional normal range	95 (23)	34 (25)	61 (22)	0.320

Abbreviations: ALP, Alkaline phosphatase; CRP, C‐reactive protein; IMDC, International Metastatic Renal Cell Carcinoma Database Consortium; NLR, nutrophil lymphocyto ratio.

### 3.2 Antitumor effects

**FIGURE 1:**
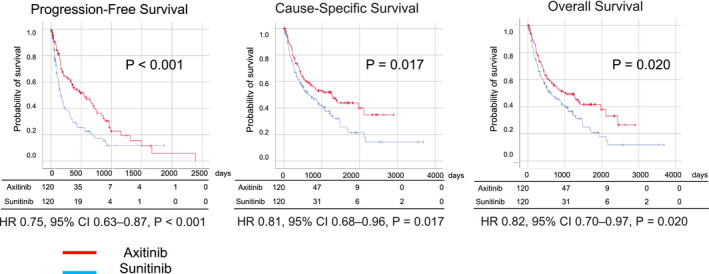
Kaplan–Meier curve of progression‐free survival, cause‐specific survival, and overall survival after propensity score matching analysis in mRCC patients treated with axitinib or sunitinib as the first‐line treatment

**FIGURE 2:**
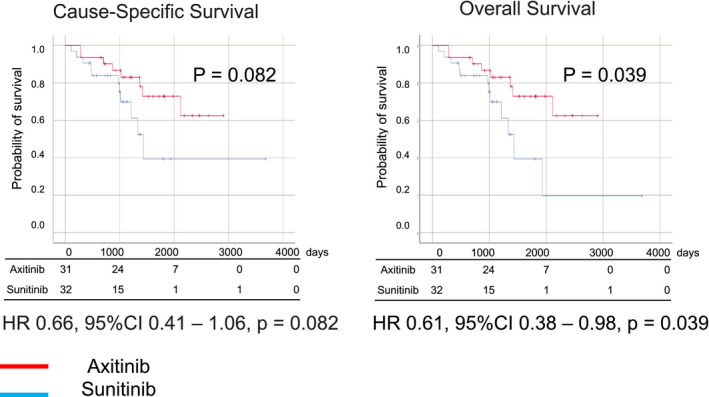
Kaplan–Meier curve of cause‐specific survival and overall survival after propensity score matching analysis in mRCC patients treated by nivolumab as sequential therapy after axitinib or sunitinib treatment

An objective response was noted in 34 and 50 patients in the axitinib and sunitinib groups, respectively (25.4% vs. 18.2%, OR: 0.657, 95% CI: 0.401–1.074, *p* = 0.095) (Table 2). A better disease control rate was achieved with axitinib than with sunitinib (73.1% vs. 62.8%, OR: 0.619, 95% CI: 0.394–0.973, *p* = 0.038). The survival outcomes were also better in axitinib group than sunitinib group in terms of PFS (HR 0.75, 95% CI 0.63–0.87, *p* < 0.001), CSS (HR 0.81, 95% CI 0.68–0.96, *p* = 0.017), and OS (HR 0.82, 95% CI 0.70–0.97, *p* = 0.020) (Figure 1). Furthermore, we compared patients treated with axitinib and sunitinib after propensity score matching (The results of PFS, CSS, and OS in all patients are shown in Figure S2). Propensity scores were calculated for each patient using multivariate logistic regression analysis with sex, age, histology, prior nephrectomy, history of nivolumab treatment, clinical stage at the first diagnosis, and the IMDC score (Table 3). The objective response rate (26.4% vs. 14.0%, OR: 0.46, 95% CI: 0.24–0.87, *p* = 0.016), disease control rate (74.4% vs. 56.2%, OR: 0.44, 95% CI: 0.26–0.76, *p* = 0.003), PFS (18.0 months vs. 5.5 months, HR: 0.75, 95% CI: 0.63–0.87, *p* < 0.001), CSS (41.9 months vs. 22.0 months, HR: 0.81, 95% CI: 0.68–0.96, *p* = 0.017), and OS (33.5 months vs. 19.8 months, HR: 0.82, 95% CI: 0.70–0.97, *p* = 0.020) were significantly better in the axitinib group than in the sunitinib group (Table 4). The propensity score matching analysis showed better OS among the patients treated with nivolumab sequentially in the axitinib group (75.1 months vs. 56.1 months, HR: 0.61, 95% CI: 0.38–0.98, *p* = 0.039; Figure 2).

**TABLE 2:** Treatment outcome of axitinib or sunitinib therapy for mRCC

	All patients (*N* = 408)	Axitinib (*N* = 134)	Sunitinib (*N* = 274)	*p*
Observational period from first‐line therapy (months)				
Median	20	20	20	0.868
Range	1–144	1–95	1–144	
Treatment duration of first‐line therapy (months)				
Median	5	8	5	0.030
Range	1–34	1–79	1–93	
Objective response, *n* (%)				
	84 (21)	34 (25)	50 (18)	0.095
Disease control, *n* (%)				
	270 (67)	98 (73)	172 (63)	0.038
Best response, *n* (%)				
CR	3 (1)	2 (1)	1 (0)	
PR	81 (20)	32 (24)	49 (17)	
SD	186 (46)	64 (48)	122 (45)	
PD	109 (27)	33 (25)	76 (57)	
Not assessed	12 (3)	3 (2)	26 (19)	

Abbreviations: CR, complete remission; mRCC, metastatic renal cell carcinoma; PD, progressive disease; PR, partial response; SD, stable disease.

**TABLE 3:** Patient characteristics after PS matching

	Axitinib (*N* = 121)	Sunitinib (*N* = 121)	OR	95% CI	*p*
Gender					
Male	90	86	0.846	0.482–1.487	0.665
Female	31	35			
Age					
	67 (33–87)	67 (33–82)			0.650
Histology					
Clear cell	100	97	0.848	0.446–1.614	0.741
Others	21	24			
Prior nephrectomy					
Yes	78	80	1.076	0.635–1.823	0.893
Nivolumab sequential					
Yes	31	32	1.044	0.590–1.848	1.000
Clinical stage at the time of diagnosis with RCC					
1	10	10			0.858
2	11	7			
3	22	22			
4	78	79			
IMDC risk classification					
Favorable	8	7			0.811
Intermediate	49	47			
Poor	48	54			

Abbreviations: IMDC, International Metastatic Renal Cell Carcinoma Database Consortium; PS, propensity score; RCC, renal cell carcinoma.

**TABLE 4:** Treatment outcome after PS matching

	All patients (*N* = 242)	Axitinib (*N* = 121)	Sunitinib (*N* = 121)	OR	95% CI	*p*
Observational period from first‐line therapy (months)						
Median	17	23	15			0.018
Range	1–121	1–95	1–121			
Treatment duration of forst‐line therapy (months)						
Median	5	9	3			<0.001
Range	1–79	1–79	1–61			
Objective response, *n* (%)						
	49 (20)	32 (26)	17 (14)	0.455	0.238–0.868	0.016
Disease control, *n* (%)						
	158 (65)	90 (74)	68 (56)	0.442	0.257–0.759	0.003
Best response, *n* (%)						
CR	2 (1)	2 (2)	0 (0)			0.372
PR	47 (19)	30 (25)	17 (14)			
SD	109 (45)	58 (48)	51 (42)			
PD	70 (29)	28 (23)	42 (35)			
Not assessed	14 (6)	3 (2)	11 (9)			

Abbreviations: CI, confidence interval; CR, complete remission; mRCC, metastatic renal cell carcinoma; OR, odds ratio; PD, progressive disease; PR, partial response; SD, stable disease.

## 4 DISCUSSION

In this retrospective study, axitinib showed a clear survival benefit over sunitinib after propensity score matching. Although axitinib was not superior to sunitinib in terms of OS when administered as first‐line therapy for mRCC in the phase III study,^13^ some retrospective studies suggested that axitinib afforded better clinical outcomes.^14^, ^15^, ^16^ The good efficacy shown by axitinib in real clinical settings might be explained by the higher relative dose intensity achieved in the axitinib group than in the sunitinib group and the greater use of the drug in many elderly patients than in clinical trials. A significantly higher rate of toxicity‐related discontinuation was observed in the sunitinib group than in the axitinib group.^16^, ^17^ Since the recommended sunitinib dose could be intolerable, especially in Asians, many physicians prefer to initiate sunitinib starting from a dose of 37.5 mg.^18^ Additionally, the lasting effect of sunitinib‐related toxicities is an unignorable factor related to poor prognoses.^19^ An appropriate first‐line therapy could affect later‐line treatment outcomes. In a previous study, the progressive disease (PD) rate for second‐line therapy was significantly higher in the first‐line sunitinib group (52%) than that in the first‐line axitinib group (26%), although there was no significant difference in the second‐line regimens between the groups.^16^ These results may suggest that appropriate first‐line therapy allows for better oncological outcomes.

Thus far, VEGFR‐TKI monotherapy has not been employed as the first‐line option for mRCC because IO combinations are preferable treatments for patients with all risk classifications of IMDC.^20^, ^21^ Nivolumab plus ipilimumab showed a complete response (CR) rate of 10% in intermediate‐ and poor‐risk patients,^4^ and pembrolizumab plus lenvatinib achieved a CR rate of 19% in patients at all varying degrees of risk.^22^ However, approximately 20% of the patients treated with IO combinations had a primary refractory status.^4^ In addition, patients with mRCC could acquire treatment resistance.^23^ These patients need second‐ or later‐line therapy, and VEGFR‐TKIs are the main options in these situations.^9^ Although the National Comprehensive Cancer Network guidelines recommend four VEGFR‐TKIs as category 1 drugs,^24^ it is not known which drug is preferable as sequential therapy. In the last 5 years, cabozantinib has been considered the main second‐line therapy after IO drugs, especially after IO plus axitinib therapy.^25^ Nevertheless, axitinib and sunitinib are among the main VEGFR‐TKI options for treating patients with mRCC. To preserve cabozantinib as a later‐line therapy should be a feasible option because cabozantinib is the only VEGFR‐TKI to have shown efficacy after other VEGFR‐TKI failures.^26^ Which drugs are better as second‐ or later‐line therapies in the IO era remains to be elucidated. Unfortunately, most studies do not provide this crucial information.^15^, ^16^ In this study, 26.5% of the patients were administered with nivolumab in sequential therapy. There should raise an argument that nivolumab was only administered as second‐ or later‐line therapy in this study. The time of initiation of IO drug treatment might not be a major concern. The KEYNOTE‐426 trial has shown better OS with pembrolizumab plus axitinib than with sunitinib.^5^ However, in this study, fewer patients were given IO drugs in the sunitinib group after sunitinib failure (23.1% at most). On the other hand, although in the JAVELIN Renal 101 trial, significant OS was not achieved, patients in the sunitinib group received more IO drugs (33.2% at most) as sequential therapy.^6^ Other first‐line IO combination therapies, which showed treatment efficacy of them, also relatively low sequential IO therapy after sunitinib failure as low as the KEYNOTE‐426.^4^, ^22^, ^27^ This might suggest that the choice of the IO drug may not depend on the treatment order.

This study has several limitations. First, we were unable to control for selection bias and other unmeasurable confounders because of the retrospective nature of the study. Second, information about the adverse events in the cohort was lacking. Third, there may be a regional bias, and our results may not be generalizable in other populations owing to differences in medical practices. Nevertheless, to the best of our knowledge, this is the first study to report the favorable oncological outcomes of axitinib in the IO era. Further research is warranted to address the clinical benefit of the axitinib in patients with mRCC.

## 5 CONCLUSIONS

First‐line axitinib therapy showed better efficacy and survival benefit for patients with mRCC than did sunitinib. Thus, axitinib might be a possible option from among the VEGFR‐TKIs available for application during IO drug‐based treatment sequences.

## CONFLICT OF INTERESTS

The authors declare no potential conflicts of interest.

## Supporting information

Fig S1

Fig S2

Table S1

## Data Availability

The data that support the findings of this study are available from the corresponding author upon reasonable request.

## References

[1] Sun M, Thuret R, Abdollah F, et al. Age‐adjusted incidence, mortality, and survival rates of stage‐specific renal cell carcinoma in North America: a trend analysis. Eur Urol. 2011;59:135‐141. 21035250 10.1016/j.eururo.2010.10.029

[2] Motzer RJ, Hutson TE, Tomczak P, et al. Sunitinib versus interferon alfa in metastatic renal‐cell carcinoma. N Engl J Med. 2007;356:115‐124. 17215529 10.1056/NEJMoa065044

[3] Rini BI, Escudier B, Tomczak P, et al. Comparative effectiveness of axitinib versus sorafenib in advanced renal cell carcinoma (AXIS): a randomised phase 3 trial. Lancet. 2011;378:1931‐1939. 22056247 10.1016/S0140-6736(11)61613-9

[4] Motzer RJ, Tannir NM, McDermott DF, et al. Nivolumab plus Ipilimumab versus Sunitinib in advanced renal‐cell carcinoma. N Engl J Med. 2018;378:1277‐1290. 29562145 10.1056/NEJMoa1712126 PMC5972549

[5] Rini BI, Plimack ER, Stus V, et al. Pembrolizumab plus axitinib versus sunitinib for advanced renal‐cell carcinoma. N Engl J Med. 2019;380:1116‐1127. 30779529 10.1056/NEJMoa1816714

[6] Motzer RJ, Penkov K, Haanen J, et al. Avelumab plus axitinib versus sunitinib for advanced renal‐cell carcinoma. N Engl J Med. 2019;380:1103‐1115. 30779531 10.1056/NEJMoa1816047 PMC6716603

[7] Motzer RJ, Escudier B, McDermott DF, et al. Survival outcomes and independent response assessment with nivolumab plus Ipilimumab versus Sunitinib in patients with advanced renal cell carcinoma: 42‐month follow‐up of a randomized phase 3 clinical trial. J Immunother Cancer. 2020;8:e000891. 32661118 10.1136/jitc-2020-000891 PMC7359377

[8] Hasanov E, Gao J, Tannir NM. The immunotherapy revolution in kidney cancer treatment: scientific rationale and first‐generation results. Cancer J. 2020;26:419‐431. 32947310 10.1097/PPO.0000000000000471

[9] Mori K, Schmidinger M, Quhal F, Egawa S, Shariat SF, Grunwald V. What is next in second‐ and later‐line treatment of metastatic renal cell carcinoma? review of the recent literature. Curr Opin Urol. 2021;31(3):276‐284. 33742984 10.1097/MOU.0000000000000867

[10] Konishi S, Hatakeyama S, Numakura K, et al. Validation of the IMDC prognostic model in patients with metastatic renal‐cell carcinoma treated with first‐line axitinib: a multicenter retrospective study. Clin Genitourin Cancer. 2019;17:e1080‐e1089. 31416753 10.1016/j.clgc.2019.07.006

[11] Santoni M, Buti S, Conti A, et al. Prognostic significance of host immune status in patients with late relapsing renal cell carcinoma treated with targeted therapy. Target Oncol. 2015;10:517‐522. 25559290 10.1007/s11523-014-0356-3

[12] Kume H, Kakutani S, Yamada Y, et al. Prognostic factors for renal cell carcinoma with bone metastasis: who are the long‐term survivors? J Urol. 2011;185:1611‐1614. 21419440 10.1016/j.juro.2010.12.037

[13] Hutson TE, Al‐Shukri S, Stus VP, et al. Axitinib versus sorafenib in first‐line metastatic renal cell carcinoma: overall survival from a randomized phase III trial. Clin Genitourin Cancer. 2017;15:72‐76. 27498023 10.1016/j.clgc.2016.05.008

[14] Rousseau B, Kempf E, Desamericq G, et al. First‐line antiangiogenics for metastatic renal cell carcinoma: a systematic review and network meta‐analysis. Crit Rev Oncol Hematol. 2016;107:44‐53. 27823651 10.1016/j.critrevonc.2016.08.012

[15] Tamada S, Iguchi T, Kato M, Yasuda S, Yamasaki T, Nakatani T. Second‐line treatment after sunitinib therapy in patients with renal cell carcinoma: a comparison of axitinib and mammalian target of rapamycin inhibitors. Oncotarget. 2018;9:37017‐37025. 30651932 10.18632/oncotarget.26439 PMC6319347

[16] Konishi S, Hatakeyama S, Tanaka T, et al. Comparison of axitinib and sunitinib as first‐line therapies for metastatic renal cell carcinoma: a real‐world multicenter analysis. Med Oncol. 2018;36:6. 30474747 10.1007/s12032-018-1231-3

[17] Numakura K, Fujiyama N, Takahashi M, et al. Clinical implications of pharmacokinetics of sunitinib malate and N‐desethyl‐sunitinib plasma concentrations for treatment outcome in metastatic renal cell carcinoma patients. Oncotarget. 2018;9:25277‐25284. 29861870 10.18632/oncotarget.25423 PMC5982748

[18] Numakura K, Tsuchiya N, Kagaya H, et al. Clinical effects of single nucleotide polymorphisms on drug‐related genes in Japanese metastatic renal cell carcinoma patients treated with sunitinib. Anticancer Drugs. 2017;28:97‐103. 27564227 10.1097/CAD.0000000000000425

[19] Guida FM, Santoni M, Conti A, et al. Alternative dosing schedules for sunitinib as a treatment of patients with metastatic renal cell carcinoma. Crit Rev Oncol Hematol. 2014;92:208‐217. 25151214 10.1016/j.critrevonc.2014.07.006

[20] Albiges L, Powles T, Staehler M, et al. Updated European Association of Urology Guidelines on renal cell carcinoma: immune checkpoint inhibition is the new backbone in first‐line treatment of metastatic clear‐cell renal cell carcinoma. Eur Urol. 2019;76:151‐156. 31151678 10.1016/j.eururo.2019.05.022

[21] Numakura K, Kobayashi M, Muto Y, et al. First‐line axitinib therapy is less effective in metastatic renal cell carcinoma with spindle histology. Sci Rep. 2020;10:20089. 33208816 10.1038/s41598-020-77135-6 PMC7675987

[22] Motzer R, Alekseev B, Rha SY, et al. Lenvatinib plus pembrolizumab or everolimus for advanced renal cell carcinoma. N Engl J Med. 2021;384:1289‐1300. 33616314 10.1056/NEJMoa2035716

[23] Mollica V, Di Nunno V, Gatto L, et al. Resistance to systemic agents in renal cell carcinoma predict and overcome genomic strategies adopted by tumor. Cancers (Basel). 2019;11(6):830. 10.3390/cancers11060830 PMC6627706 31207938

[24] Motzer RJ, Jonasch E, Michaelson MD, et al. NCCN guidelines insights. kidney cancer, version 2.2020. J Natl Compr Canc Netw. 2019;17(11):1278‐1285. 31693980 10.6004/jnccn.2019.0054

[25] McGregor BA, Lalani AA, Xie W, et al. Activity of cabozantinib after immune checkpoint blockade in metastatic clear‐cell renal cell carcinoma. Eur J Cancer. 2020;135:203‐210. 32599410 10.1016/j.ejca.2020.05.009

[26] Choueiri TK, Escudier B, Powles T, et al. Cabozantinib versus Everolimus in advanced renal‐cell carcinoma. N Engl J Med. 2015;373:1814‐1823. 26406150 10.1056/NEJMoa1510016 PMC5024539

[27] Choueiri TK, Powles T, Burotto M, et al. Nivolumab plus Cabozantinib versus Sunitinib for advanced renal‐cell carcinoma. N Engl J Med. 2021;384:829‐841. 33657295 10.1056/NEJMoa2026982 PMC8436591

