# Supplementary material for: Outcomes of axitinib versus sunitinib as first‐line therapy to patients with metastatic renal cell carcinoma in the immune‐oncology era
Source: Cancer Med. 2021 Jul 27;10(17):5839–46. doi: 10.1002/cam4.4130 (PMC8419787; doi:10.1002/cam4.4130)
Supplement: Supplementary file 1 — Fig S1 [file CAM4-10-5839-s003.pdf]

Supplement Figure 1. Selection of patients. Of 703 patients with mRCC, we selected 408 patients who were treated with axitinib or sunitinib as first-line VEGFR-TKIs in this study.

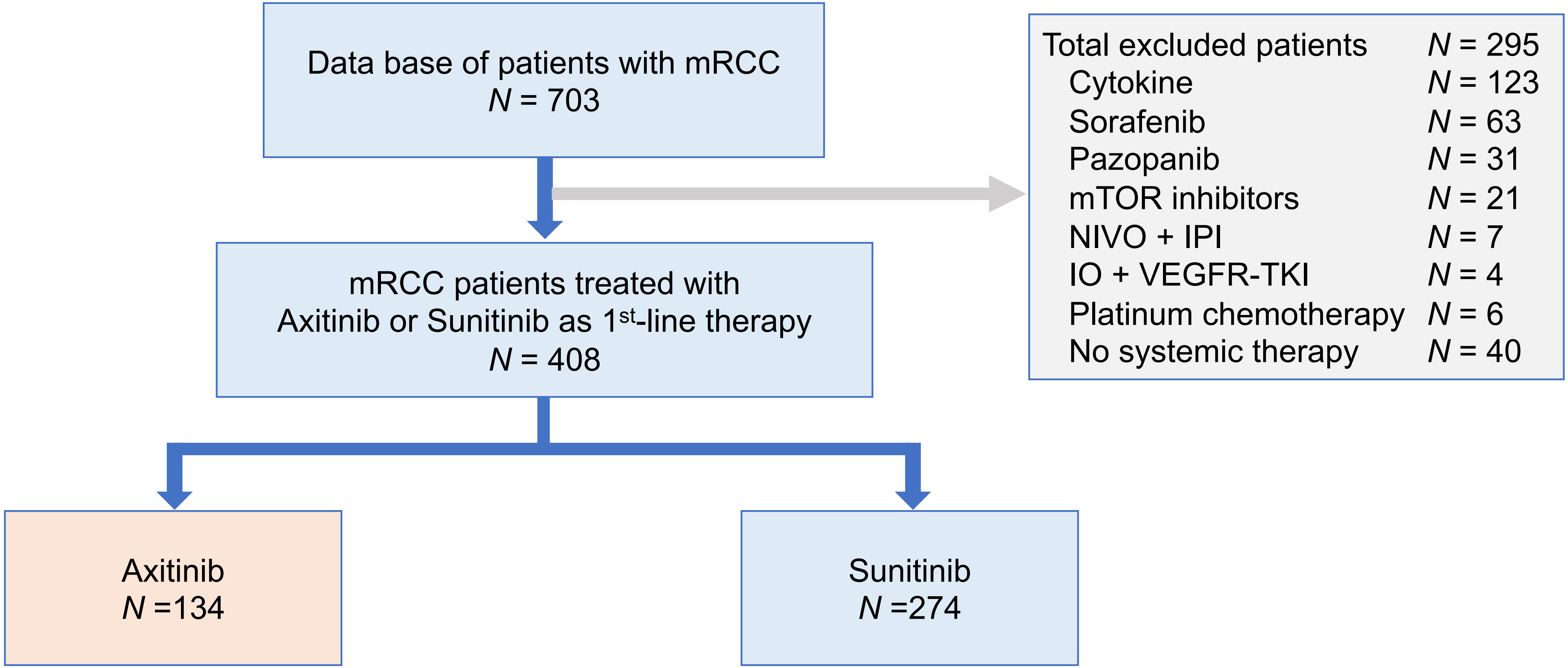

mRCC, metastatic renal cell carcinoma; VEGFR, vascular endothelial growth factor receptor; TKI, tyrosine kinase inhibitor; mammalian target of rapamycin; NIVO, nivolumab; IPI, ipilimumab, IO, immuno-oncology drug
