# Supplementary material for: Outcomes of axitinib versus sunitinib as first‐line therapy to patients with metastatic renal cell carcinoma in the immune‐oncology era
Source: Cancer Med. 2021 Jul 27;10(17):5839–46. doi: 10.1002/cam4.4130 (PMC8419787; doi:10.1002/cam4.4130)
Supplement: Supplementary file 2 — Fig S2 [file CAM4-10-5839-s002.pdf]

Supplement Figure 2. Kaplan–Meier curve of progression-free survival, cause-specific survival, and overall survival in all mRCC patients treated with axitinib or sunitinib as the first-line treatment

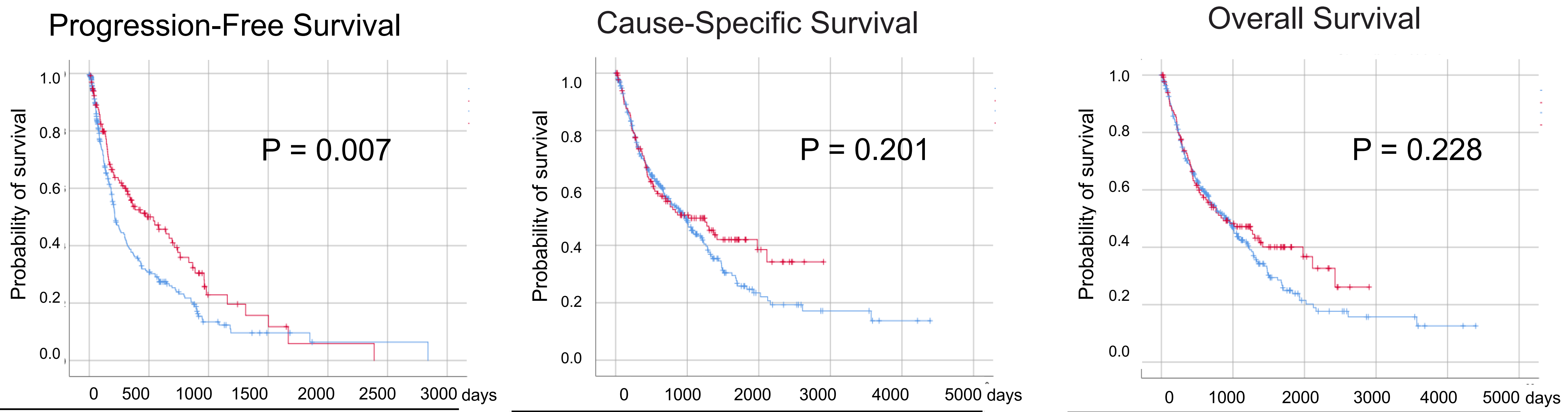

Progression-Free Survival

Axitinib	133	36	7	4	1	0	0
Sunitinib	270	57	13	4	1	1	0

HR 0.83, 95% CI 0.73–0.95, P = 0.007

Cause-Specific Survival

Axitinib	134	48	10	0	0	0
Sunitinib	273	86	17	6	2	0

HR 0.91, 95% CI 0.79–1.05, P = 0.201

Overall Survival

Axitinib	134	48	10	0	0	0
Sunitinib	273	86	17	6	2	0

HR 0.92, 95% CI 0.80–1.05, P = 0.228

Axitinib
Sunitinib
